# Longitudinal Analysis of Patient-Reported Outcomes in Clinical Trials: Applications of Multilevel and Multidimensional Item Response Theory

**DOI:** 10.1007/s11336-021-09777-y

**Published:** 2021-06-17

**Authors:** Li Cai, Carrie R. Houts

**Affiliations:** 1 University of California, 300 Charles E. Young Dr. N, 315 GSEIS Bldg., Los Angeles, CA 90095-1522 USA; 2 Vector Psychometric Group, LLC, Durham, USA

**Keywords:** item response theory, multilevel modeling, growth modeling

## Abstract

With decades of advance research and recent developments in the drug and medical device regulatory approval process, patient-reported outcomes (PROs) are becoming increasingly important in clinical trials. While clinical trial analyses typically treat scores from PROs as observed variables, the potential to use latent variable models when analyzing patient responses in clinical trial data presents novel opportunities for both psychometrics and regulatory science. An accessible overview of analyses commonly used to analyze longitudinal trial data and statistical models familiar in both psychometrics and biometrics, such as growth models, multilevel models, and latent variable models, is provided to call attention to connections and common themes among these models that have found use across many research areas. Additionally, examples using empirical data from a randomized clinical trial provide concrete demonstrations of the implementation of these models. The increasing availability of high-quality, psychometrically rigorous assessment instruments in clinical trials, of which the Patient-Reported Outcomes Measurement Information System (PROMIS®) is a prominent example, provides rare possibilities for psychometrics to help improve the statistical tools used in regulatory science.

## Introduction

After years of research and continued investment, patient-reported outcomes (PROs) have emerged as important outcomes in clinical research and studies, especially as regulators, such as the US Food and Drug Administration (FDA), are increasingly focused on ensuring that patient-centered outcomes are used in clinical trials to evaluate the usefulness of drugs and medical devices (e.g., FDA, 2020). The National Institutes of Health (NIH)’s Patient-Reported Outcomes Measurement Information System (PROMIS®) initiative (NIH, n.d.) and the resulting measures have begun impacting clinical trial design and data analysis. Furthermore, there is increasing use of PROs in observational studies. A search using keywords that include “patient reported outcomes” and “observational studies” on PubMed.gov returns a 53-fold increase in the number of relevant articles from 2000 to 2019. The proliferation of PROs also brings interesting statistical modeling challenges and opportunities for readers of *Psychometrika*, particularly because of the penetration of item response theory (IRT) in PRO development and applications, together with typical clinical trial design features such as patient recruitment in multiple sites, use of randomization, and multiple follow-ups.

The typical use of PROs in a clinical trial involves the calculation of individual patients’ scores on the PRO measurement instrument. Traditionally, these are often summed scores. That has changed since PROMIS was developed. The rigorous psychometric work that has gone into the development of the PROMIS family of measures offers researchers the possibility of obtaining response pattern-based IRT-scaled scores (e.g., *expected a posteriori* [EAP] scores) using item parameters calibrated in large, representative national samples. For convenience, PROMIS has also provided summed score to EAP conversion tables to mimic full response pattern scoring, provided that all items for a given individual have no missing responses. Those pre-calculated scores then serve as the dependent variable, and important information such as treatment assignment and other variables of interest is entered into the model on the predictor side. Regardless of how the scores are obtained (summed, response pattern, summed-to-EAP conversion), a typical clinical trial analysis of such PRO data involves the examination of changes (between Baseline and end of treatment, or other follow-up periods) by randomized treatment assignment, and/or via the modeling of the outcomes directly with a general or generalized linear (or mixed) model, controlling for other covariates. In brief, the standard approach involves (1) psychometrically derived and scored outcomes and (2) the modeling of those outcome variables using standard statistical models. Importantly, the two parts are distinct, procedurally and often organizationally, with data management teams and biostatisticians handling each part (score derivation and analysis) separately.

In our view, however, the treatment of PROMIS scores as yet another set of observed variables fed into the tried-and-true regression models developed for single-variable, routine clinical trial outcomes such as heart rate, blood pressure, and other biological markers, discards many interesting and informative methods that could be applied to examine treatment effectiveness in a broad, multilevel, and multidimensional IRT modeling framework. First, unlike biological or physiological measures, the PROMIS measures are psychometrically validated, multi-item instruments. The calibrated item parameters, along with the built-in linking ability of the IRT models, provide crucial reference points with which comparability between and within studies can be achieved, thereby substantially improving cumulative science and replication. Second, while IRT can handle measurement error and improve precision, its benefit is maximized if the model simultaneously includes the necessary regression model parameters to take into account clinical trial design. This is a well-known statistical consideration, going as far back as Lindley and Smith (1972), at a minimum, who distinguished between unconditional and conditional exchangeability in Bayesian analysis. Similar ideas have also been applied in the large-scale educational assessment field (Mislevy, 1991), where latent regression models with multidimensional IRT on the outcome side of the regression equation have, for decades, supported unbiased population inference in assessment programs such as the National Assessment of Education Progress (NAEP). Such latent regression models assume the existence of calibrated items that can define the location and scale of the student achievement/proficiency variables of interest, akin to the calibrated PROMIS item banks. Mislevy et al. (1992) noted the paradoxical result that seriously biased population inference can result even from individually optimal latent proficiency estimates (such as IRT scaled scores), with the same paradox also arising in the estimation of change (p. 137). The IRT calibration can of course be included in a joint estimation approach along with the estimation of population /regression parameters, but the benefit of leaving IRT-based item calibration as a separate step is substantial simplicity of procedure and the possibility of leveraging results from existing large-sample calibrations to randomized or quasi-experimental studies having much smaller *N*-count. Therefore, if individual scores are never produced and marginal inference procedures such as the approach advocated here are adopted, one can obtain consistent estimates of population characteristics even when the sample size in the analysis may not fully support the simultaneous estimation of all item parameters and latent regression parameters.

Third, over the past decade or more, multidimensional and multilevel IRT models have developed to such an extent, including its statistical theory and software readiness, that typical research questions in clinical trial settings can be implemented directly within multilevel and multidimensional IRT. Finally, the proposed integrative analytical approach combines methods popular in psychometrics, largely out of discrete multivariate analysis, with methods popular in biometrics, largely out of generalized linear and mixed effects modeling. The intersection of these two often-disparate fields potentially yields more flexible and powerful methods to understand treatment effectiveness in randomized controlled trials (see, e.g., Cai et al., 2016).

The main purpose of the current work is to demonstrate empirically practicable latent variable analyses that may be informative for understanding change over time in PRO data from clinical trials. In the process of this modeling exploration, we will highlight connections between various models and, in the analysis section, provide practical demonstrations of models and relationships and equivalences among methods previously described (e.g., Bock & Bargmann, 1966; Cai et al., 2016; Curran et al., 2008; Curran, 2003; Embretson, 1991; MacCallum et al., 1997; McArdle, 2009; Paek et al., 2014).

## The Motivating Data Set

The data analyzed stems from a previously completed phase-2 clinical trial. The data were collected from a multicenter, individually randomized, double-blind, placebo-controlled study. The specific disease area and compound are withheld as the full trial results have not yet been made public. Participants were randomly assigned to either the placebo or active treatment condition.

**Table 1:** PROMIS short form V.1.0-sleep disturbance 8a T-scores descriptive statistics by treatment group and visit.

Treatment group	Baseline			Follow-up 1			Follow-up 2		
	*n*	*M*	(SD)	*n*	*M*	(SD)	*n*	*M*	(SD)
Placebo	137	60.81	(8.56)	133	55.08	(8.46)	126	53.02	(9.20)
Treatment group	112	61.79	(7.97)	107	50.88	(10.60)	103	48.54	(10.66)

**Fig. 1:**
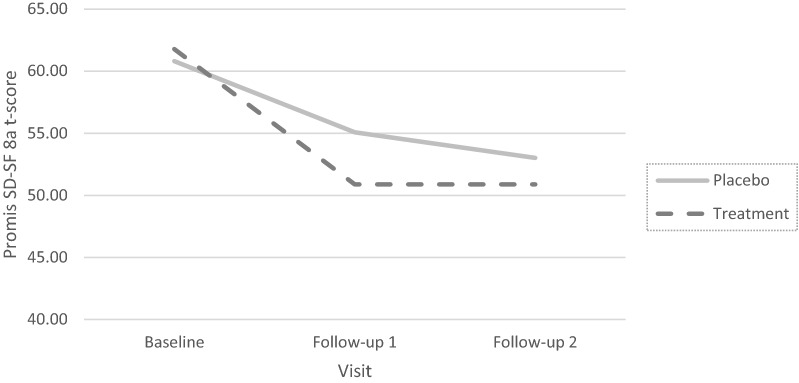
PROMIS Short Form V.1.0-Sleep disturbance 8a T-scores by visit and treatment group

Sleep disturbance (SlpDist), though not the primary endpoint, was measured and studied here, given the known significant relation between the disease of interest and sleep issues. The PROMIS Short Form V.1.0-Sleep Disturbance 8a (Yu et al., 2011), an 8-item self-report measure, was used in the trial. All items use a 7-day recall period and are answered using a 5-category ordinal response scale (1: Very poor/ Not at all, to 5: Very good/Very much, depending on item content). In the current analyses, item responses from assessments at three visits will be analyzed: Baseline, Follow-up 1, and Follow-up 2 (end of treatment). The total baseline sample size for this study is 249. One participant does not have baseline data to analyze, but is present in the follow-up data, making the unique number of cases 250. We elected to include the particular case because all models described here can handle missing observations. The PROMIS Short Form V.1.0-Sleep Disturbance 8a scores are derived from item parameters based on large, national calibration samples. The scores are reported on the PROMIS *T*-score metric, which has a population mean of 50 and standard deviation (SD) of 10; to add in score interpretation, it is useful to know that in the SlpDist item bank, the sample used to calibrate the items was a mixture of participants from the US general population and a clinical sample (Buysse et al., 2010). Basic descriptive information of the PROMIS Short Form V.1.0-Sleep Disturbance 8a scores T-scores (found by conversion from summed scores) by treatment assignment and visit is reported in Table 1 and Fig. 1; no individual assessment had item-level missingness, making these values acceptable to use.

## Notation and Models

The notation and models described here are simplified version of Cai et al.’s (2016) general multilevel multidimensional item response theory framework, but without much of their added complexities due to the presence of discrete latent variables to accommodate diagnostic classification models (e.g., von Davier & Lee, 2019) in their framework. We will first examine the latent structural models, including the specification of regression parameters, before adding the nonlinear link functions of IRT to complete the specification of measurement models. Finally, the structural and measurement models will be combined with the help of the conditional independence assumption.

In a non-trivial manner, the models presented here draw their inspirations from how NAEP dealt with the challenge of unbiased aggregated population inference by combining measurement error-prone individual-level item response data with background covariates in a regression model, wherein the outcome variables are latent (e.g., Mislevy et al., 1992). Interested readers are referred to the special issue edited by Cai (2019) for further details. The main differences are trifold. First, NAEP conducts its own item calibration before using the item parameter estimates in the latent variable regression, whereas PROMIS measures arrive already IRT-calibrated. This is convenient for clinical researchers because not all clinical trials have the sample size needed for stable IRT model calibration. Second, NAEP does not emphasize the interpretation of the latent regression parameters, but in our analysis, those parameters are critical. Third, the NAEP machinery aims at producing institutionally generated multiple imputations in the form of plausible values so that researchers external to NAEP could model the data on their own. Schofield et al. (2015) contain more discussions on the use of NAEP-provided scores. In contrast, our models emphasize likelihood-based inference for regression coefficients and variance components that carry substantive meaning, just as in the application of a standard regression analysis, mixed models, or growth curve models for clinical trial data.

### The Latent Structural Model

#### Back to Basics

Consider the situation where $\eta _{ijt}$ represents a latent outcome variable of interest (e.g., depression, physical functioning, or sleep disturbance). The subscripts indicate that this is a value from site *i*, individual $j=1,\ldots J_{i}$ in that site, and at occasion $t=1,\ldots ,T$. For the moment, let us omit the site subscript (to be added back in Sect. 3.1.3). With this simplification, for individual *j*, the following multivariate regression model should be a familiar sight:

1 $$\begin{aligned} \left( {\begin{array}{*{20}c} \eta _{j1}\\ \vdots \\ {\begin{array}{*{20}c} \eta _{jt}\\ \vdots \\ \eta _{jT}\\ \end{array} }\\ \end{array} } \right) =\varvec{\eta }_{j}= {\mathbf {{B}}}{{{{\varvec{x}}}}}_{j}+\varvec{\epsilon }_{j}, \end{aligned}$$

where ${{{{\varvec{x}}}}}_{j}$ is a vector of fixed predictor values (e.g., dummy variables coding the cells of the experimental design), ${\mathbf {{B}}}$ contains the matrix of regression coefficients, and $\varvec{\epsilon }_{j}$ is the error term with mean 0 and a typically unstructured covariance matrix.

When the observations are fully stacked, as is typical in a multivariate regression model, statistical modelers of repeated-measures data should immediately recognize Eq. (1) as synonymous to the multivariate linear model used in the multivariate approach to repeated-measures ANOVA

2 $$\begin{aligned} \left( {\begin{array}{*{20}c} \varvec{\eta }_{1}^{'}\\ \vdots \\ {\begin{array}{*{20}c} \varvec{\eta }_{j}^{'}\\ \vdots \\ \varvec{\eta }_{J}^{'}\\ \end{array} }\\ \end{array} } \right) ={\mathbf {{H}}}={\mathbf {{XB}}}+{\mathbf {{E}}}, \end{aligned}$$

where ${{\mathbf {X}}}$ is the design matrix, and the rows of ${\mathbf {{E}}}$ are assumed independent. When all values in ${\mathbf {{H}}}$ are observable quantities, the linear least squares fitting of the model in Eq. (2) to data require minimal assumptions or computational effort. With the additional (matrix) Gaussian distribution assumptions on the error term ${\mathbf {{E}}}$, classical hypothesis testing of various between- and within-subject effects is routinely available (see, e.g., Mardia et al., 1979).

#### Adding Random Effects

Unfortunately, in our setting, where elements of ${\mathbf {{H}}}$ are latent, this classical multivariate analysis of variance model is not directly applicable. Furthermore, the advent of powerful linear mixed effects models (also called multilevel or hierarchical linear models) as well as software packages afford the modeler increased flexibility over traditional linear models (e.g., Searle et al., 1992). We now briefly return to the univariate case and choose to model the outcome $\eta _{jt}$ using a combination of fixed and random effects, realizing that the random effects *are* latent variables after all (e.g., Bauer, 2003; Curran, 2003).

The concept and notation popularized by Raudenbush and Bryk’s (2002) hierarchical linear modeling textbook may be particularly useful here: individuals may have different initial status and may also have different rates of change. Both may be randomly varying over a population of individuals. To model the effect of time, a simple linear time code can be inserted into the model as $x(t)=t-1$, though more complex variants exist (see, e.g., MacCallum et al., 1997 and references therein). The random intercept coefficient $\theta _{0j}$ represents the individual variability in outcomes at baseline, and $\theta _{1j}$ is the random slope coefficient:

3 $$\begin{aligned} \eta _{jt}=\theta _{0j}+\theta _{1j}x(t)+\epsilon _{jt}. \end{aligned}$$

When there are no explanatory variables for the random coefficients, we have an “unconditional” model, which may be expressed more compactly in matrix notation as

4 $$\begin{aligned} \left( {\begin{array}{*{20}c} \eta _{j1}\\ \eta _{j2}\\ \eta _{j3}\\ \end{array} } \right) =\varvec{\eta }_{j}=\left( {\begin{array}{*{20}c} 1 &{} 0\\ 1 &{} 1\\ 1 &{} 2\\ \end{array} } \right) \left( {\begin{array}{*{20}c} \theta _{0j}\\ \theta _{1j}\\ \end{array} } \right) +\left( {\begin{array}{*{20}c} \epsilon _{j1}\\ \epsilon _{j2}\\ \epsilon _{j3}\\ \end{array} } \right) =\left( {\begin{array}{*{20}c} {\theta _{0j}+0\theta _{1j}+\epsilon }_{j1}\\ \theta _{0j}+{1\theta _{1j}+\epsilon }_{j2}\\ \theta _{0j}+2\theta _{1j}+\epsilon _{j3}\\ \end{array} } \right) , \end{aligned}$$

Equation (4) makes it apparent that the model may be reformulated as a latent curve model (Meredith & Tisak, 1990), following a long tradition in psychometrics. The reader is referred to Bollen and Curran (2006) for a comprehensive treatment of this subject, as well as the equivalence between the latent curve model and mixed models for repeated-measures data.

The random coefficients $\theta _{0j}$ and $\theta _{1j}$ may be regressed on design variables and covariates. Consider the case of a single predictor $x_{j}$, e.g., the treatment assignment indicator, the pair of regression equations becomes:

5 $$\begin{aligned} \theta _{0j}= & {} \beta _{00}+\beta _{01}x_{j}{+\gamma }_{0j} \nonumber \\ \theta _{1j}= & {} \beta _{10}+\beta _{11}x_{j}{+\gamma }_{1j}, \end{aligned}$$

where the $\beta$’s are the fixed effect regression coefficients, and $\gamma$’s are the random effects. Substituting Eq. (5) into (3) and collecting terms leave us with yet another familiar sight, namely the linear mixed model for longitudinal data with so-called cross-level interactions

6 $$\begin{aligned} \eta _{jt}=\beta _{00}+x\left( t \right) \beta _{10}+x_{j}\beta _{01}+\beta _{11}x_{j}x\left( t \right) +\gamma _{0j}+x\left( t \right) \gamma _{1j}+\epsilon _{jt}. \end{aligned}$$

In our motivating example with 3 measurement occasions and two treatment groups, we see that Eq. (6) can be written more compactly in matrix form as

7 $$\begin{aligned} \left( {\begin{array}{*{20}c} \eta _{j1}\\ \eta _{j2}\\ \eta _{j3}\\ \end{array} } \right) =\varvec{\eta }_{j}=\left( {\begin{array}{*{20}c} 1&{}0&{}x_j&{}0\\ 1&{}1&{}x_j&{}x_j\\ 1&{}2&{}x_j&{}2x_j\\ \end{array} } \right) \left( {\begin{array}{*{20}c} \beta _{00}\\ \beta _{10}\\ {\begin{array}{*{20}c} \beta _{01}\\ \beta _{11}\\ \end{array} }\\ \end{array} } \right) +\left( {\begin{array}{*{20}c} 1 &{} 0\\ 1 &{} 1\\ 1 &{} 2\\ \end{array} } \right) \left( {\begin{array}{*{20}c} \gamma _{0j}\\ \gamma _{1j}\\ \end{array} } \right) +\left( {\begin{array}{*{20}c} \epsilon _{j1}\\ \epsilon _{j2}\\ \epsilon _{j3}\\ \end{array} } \right) , \end{aligned}$$

or more generally

8 $$\begin{aligned} \varvec{\eta }_{j}={{{{\varvec{X}}}}}_{j}\varvec{\beta } +{{{{\varvec{Z}}}}}_{j}\varvec{\gamma }_{j}+\varvec{\epsilon }_{j}, \end{aligned}$$

where ${{\varvec{X}}}_{j}$ and ${{\varvec{Z}}}_{j}$ are the fixed and random effects design matrix, respectively, $\varvec{\gamma }_{j}$ contains the latent variables or random effects that are typically assumed to be jointly normally distributed with zero means and a positive definite covariance matrix ${{\varvec{G}}}$, and the error term $\varvec{\epsilon }_{j}$ is uncorrelated with the random effects, with covariance matrix ${{\varvec{R}}}.$ The variance components associated with the two random effects indicate individual variability around those fixed effects, and their covariance indicates whether the rates of change are correlated with the initial status. The implied covariance matrix of $\varvec{\eta }_{j}$ is ${{\varvec{Z}}}_{j}{{\varvec{G}}}{{\varvec{Z}}}_{j}^{{\varvec{'}}}{\varvec{+R}}$, which is factor-analytic in nature.

Had the $\eta _{jt}$’s been fully observed, fitting the mixed model (or latent curve model) in Eq. (8) is now routine. A variant of Eq. (8) with an empty random effect design matrix ${{\varvec{G}}}$ but a fully unstructured covariance matrix for the error term ${{\varvec{R}}}$ is equivalent to the multivariate regression model in Eq. (2). This equivalence is important to note because it enables us to walk back-and-forth between the univariate and the multivariate approach to longitudinal data modeling and permits more versatile model specifications.

#### Multilevel and Multivariate Simultaneously

**Fig. 2:**
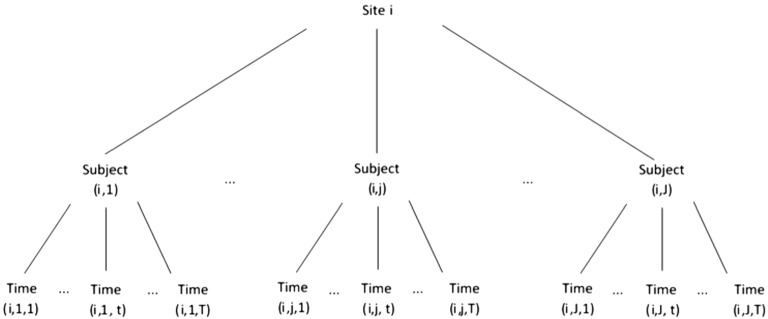
Graphical depiction of nesting seen in clinical trial patient data

Recall that we omitted the site index *i* earlier. Figure 2 provides a graphical depiction of the nesting of patients within sites and repeated measurements within patients; the individuals recruited from the same site tend to be more correlated than across sites due to this nesting. Using the study described in Sect. 2 as context, we may wish to include additional latent variables (random effects) varying at the level of sites to handle the lack of independence, such as by the addition of a site-specific random intercept. Returning to the multivariate setup where occasions are represented as additional variables, we now borrow instead from the notation used in the multilevel structural equation modeling literature (e.g., Muthén, 1994) and decompose $\varvec{\eta }_{ij}$ into a between-site random intercept ($\theta _{i})$ and a within-site component ($\varvec{\theta }_{ij})$.

9 $$\begin{aligned} \left( {\begin{array}{*{20}c} \eta _{ij1}\\ \vdots \\ {\begin{array}{*{20}c} \eta _{ijt}\\ \vdots \\ \eta _{ijT}\\ \end{array} }\\ \end{array} } \right) =\varvec{\eta }_{ij}=\theta _{i} +\varvec{\theta }_{ij}=\left( {\begin{array}{*{20}c} \theta _{i}+\theta _{ij1}\\ \vdots \\ {\begin{array}{*{20}c} \theta _{i}+\theta _{ijt}\\ \vdots \\ \theta _{i}+\theta _{ijT}\\ \end{array} }\\ \end{array} } \right) , \end{aligned}$$

In this model, the variance component related to $\theta _{i}$ indicates the extent to which there is extra site-level correlations. Obviously more complex site-level random effects may be specified. The $\varvec{\theta }_{ij}$ latent variables effectively become deviations from the site-level intercept, with their covariance matrix representing correlations in the longitudinal data, and the estimates of their means show trends over time. Equation (9) forms the core of the primary tier of latent variables in Cai’s (2010a) two-tier item factor analysis model for longitudinal item response data, but also extends it by adding latent variables to account for another level of nesting.

Equation (9) is not the only possible model that one can choose. One can adopt an alternative parameterization that draws its inspirations from the growth models laid out by Bock and Bargmann (1966), Embretson (1991), and McArdle (2009), wherein each subsequent occasion is represented by a uncorrelated latent difference:

10 $$\begin{aligned} \left( {\begin{array}{*{20}c} \eta _{ij1}\\ \vdots \\ {\begin{array}{*{20}c} \eta _{ijt}\\ \vdots \\ \eta _{ijT}\\ \end{array} }\\ \end{array} } \right) =\varvec{\eta }_{ij}=\left( {\begin{array}{l} {{\theta _i} + {\theta _{ij1}}} \\ \vdots \\ {\theta _i} + {\theta _{ij1}} + \cdots + {\theta _{ijt}} \\ \vdots \\ {\theta _i} + {\theta _{ijT}} + \cdots + {\theta _{ijt}} + \cdots + {\theta _{ijT}}\\ \end{array}} \right) . \end{aligned}$$

A favorable aspect of the specification in Eq. (10) is that the occasion-specific effects $\theta _{ijt}$ are already latent change scores, with estimable means and variances, and the site random effect is interpretable as the site-level variation at time point.

As far as the predictors are concerned, regardless of the model specification, we may regress $\varvec{\theta }_{ij}$ on individual-level predictor values contained in a vector ${{{{\varvec{x}}}}}_{ij}$

11 $$\begin{aligned} \varvec{\theta }_{ij}={{\mathbf {B}}}{{{\varvec{x}}}}_{ij}+\varvec{\epsilon }_{ij}, \end{aligned}$$

and the site random effect $\theta _{i}$ on site-level predictors ${{{{\varvec{x}}}}}_{i}$

12 $$\begin{aligned} \theta _{i}={{{{\varvec{x}}}}}_{i}^{'}\varvec{\beta }+\epsilon _{i}. \end{aligned}$$

Rather than using observed change scores, regressing the latent differences in Eq. (10) on appropriately coded predictors leads to coefficients that have clear meaning. For example, the regression of $\theta _{ijt}$ on the treatment assignment indicator $x_{j}$ (assumed to be a dummy coded variable) leads to the contrast between how much the active treatment and placebo groups changed in the outcome of interest from the prior occasion to occasion *t*, while holding site-level initial differences constant. This is effectively a latent variable version of the familiar difference-in-differences estimator widely used in econometrics for evaluating treatment effect in quasi-experimental studies (Imbens & Wooldridge, 2008).

### Multidimensional IRT Measurement Models

With the latent structural models laid out as in Sect. 3.1, we are ready to discuss the IRT-based measurement models. In principle, any multidimensional IRT model may be used, but because PROMIS measures are routinely calibrated with the logistic version of the graded response model (Samejima, 1969), which uses a cumulative logit link, we will focus on that model here. To handle the fact that the item parameters are already available and that the items are typically repeatedly administered to the patients in the clinical trial, we make two slight modifications.

In our setting, a graded response model for item $k=1,\ldots ,K$ possessing $C_{k}$ ordered categories may be expressed as the difference between two cumulative probabilities

13 $$\begin{aligned} P\left( Y_{ijkt}=c\vert \eta _{ijt},\xi _{k} \right) =P\left( Y_{ijkt}\ge c\vert \eta _{ijt},\xi _{k} \right) -P\left( Y_{ijkt}\ge c+1\vert \eta _{ijt},\xi _{k} \right) , \end{aligned}$$

where $Y_{ijkt}$ denotes the item response at occasion *t* to item *k* from individual *j* in site *i*, and

$c=0,1,\ldots ,C_{k}-1$. The cumulative probabilities are as follows:

14 $$\begin{aligned}&P\left( {{Y_{ijkt}} \ge 1|{\eta _{ijt}},{\xi _k}} \right) = {1 \over {1 + \exp \left[ { - \left( {c_{k,1}^* + a_k^*{\eta _{ijt}} + {s_k}{\xi _k}} \right) } \right] }} \nonumber \\&\vdots \nonumber \\&P\left( {{Y_{ijkt}} \ge {C_k} - 1|{\eta _{ijt}},{\xi _k}} \right) = {1 \over {1 + \exp \left[ { - \left( {c_{k,{C_k} - 1}^* + a_k^*{\eta _{ijt}} + {s_k}{\xi _k}} \right) } \right] }} \end{aligned}$$

Obviously $P\left( Y_{ijkt}\ge 0\vert \eta _{ijt},\xi _{k} \right) =1$ and $P\left( Y_{ijkt}\ge C_{k}\vert \eta _{ijt},\xi _{k} \right) =0$ are required for consistency.

**Table 2:** Unconditional random-intercept model applied to PROMIS Short Form V.1.0-Sleep disturbance 8a T-scores from the PROMIS-provided summed score to EAP conversion table.

Parameter	Est	SE	*Z* value	*p* value
*Covariance parameter estimates*				
Variance(intercept)	33.09	6.28	5.27	$< 0.0001$
Variance(slope)	12.01	3.27	3.68	$< 0.0001$
Covariance(intercept, slope)	$-$ 0.23	3.47	$-$ 0.07	0.95
Residual	35.23	3.28	10.74	$< 0.0001$

Effect	Num. DF	Den. DF	*F* value	*p* value
*Type 3 tests of fixed effects*				
Visit	1	238	220.22	$< 0.0001$
Treatment	1	228	0.04	0.84
Visit * treatment	1	228	13.92	$< 0.001$

Effect	Est	SE	*T* value	*p* value
*Solution for fixed effects*				
Intercept	60.27	0.67	89.08	$< 0.0001$
Visit	$-3.97$	0.47	$-\,8.30$	$< 0.0001$
Treatment (ref $=$ placebo)	0.21	1.01	0.21	0.84
Visit * treatment	$-\,2.67$	0.72	$-\,3.73$	$< 0.001$

Est, estimate; SE, standard error; Num. DF, numerator degrees of freedom; Den. DF, denominator degrees of freedom; Ref, reference group.

As one can see, the first modification from the standard graded model lies in the addition of an item-specific random effect $\xi _{k}$. This addition draws directly from Cai’s (2010a) two-tier item factor model for longitudinal item analysis. The items are repeatedly administered, so the extra dependence among the same item over time should be handled explicitly, or a violation of the conditional independence assumption could result. It amounts to the residual correlations found in latent curve models or in multilevel models for repeated-measures data. We shall assume the $\xi _{k}$’s to have zero means and unit variance. With the item slope of $s_{k}$ on $\xi _{k}$, we can also understand it to mean the item residual dependence variance component is $s_{k}^{2}$.

The second modification is more subtle. Instead of assuming the item parameters ($c_{k}$’s and $a_{k}$’s) have to be estimated, PROMIS has already provided banked values of intercepts and item discrimination values. We add a superscript of * to the item parameters to indicate that they are to be fixed to PROMIS item bank values. This practice mimics that of the operational procedure in NAEP, and it fully identifies the means and covariance matrices of the latent variables, particularly those of $\varvec{\eta }$. In other words, the regression models in Sect. 3.1 function almost as if the outcomes were observed, and we are back in the familiar territory of multivariate regression and analysis of variance.

As is customary in multidimensional IRT, the item responses are assumed independent conditional on all the latent variables and structural parameters in the model. Maximum marginal likelihood parameter estimation would require numerically integrating the latent variables out of the model and iterative optimization via an algorithm such as the expectation-maximization (EM) algorithm (Bock & Aitkin, 1981; Dempster et al., 1977;). Two recent methods aid computational efficiency substantially. First, the item random effects satisfy Cai’s (2010a) two-tier item factor patterns, so despite the potentially large number of items, the additional increase in the dimensionality of integration is limited because dimension reduction techniques can be applied. Second, with the increase in number of occasions, the size of $\varvec{\eta }$ necessarily increases. Stochastic optimization algorithms such the Metropolis–Hastings Robbins–Monro (MH-RM; Cai, 2010b, c) algorithm will yield considerable computational savings.

## Empirical Illustrations

### Standard Approach

As mentioned earlier, one can score the PROMIS Short Form V.1.0-Sleep Disturbance 8a with the original *T*-score conversion table. Such scores may be used as the outcome variable in a linear mixed model. We used SAS 9.4’s PROC MIXED to fit a standard random intercept, random slope growth model to the data, conditional on treatment group. Results of this analysis are presented in Table 2; a brief review finds that in addition to significant variability in intercepts and slopes across individuals, the visit, and the treatment by visit interaction were all statistically significant. As noted, however, we believe that much more interesting and useful results can leverage the psychometric work that has gone into calibrating PROMIS tools and we will also fit the data with several latent variable models previously described.

### Latent Variable Models

All latent variable models were fit in flexMIRT®3.62 (Cai, 2020) using either the Bock-Aikin EM (BAEM; Bock & Aitkin, 1981) or the MH-RM algorithm (Cai, 2010b, c). When a model was estimated with BAEM, standard errors (SEs) were estimated via the Richardson extrapolation method (e.g., Jamshidian & Jennrich, 2000). When a model was estimated with MH-RM, SEs were estimated recursively (e.g., Cai, 2010b). For all the reported models, the logistic graded response model (Samejima, 1969) item parameters for the PROMIS Short Form V.1.0-Sleep Disturbance 8a items were treated as fixed parameters, set at the values specified in the item bank.^1^ The prior psychometric validation work conducted by the PROMIS team allows us to make additional assumptions of measurement invariance over groups and time, in turn allowing for the statistical modeling to focus on studying change in the latent variables over time, and how best to associate and explain such change with available variables (such as treatment assignment). Full flexMIRT®syntax and output files for all reported models are available in the online supplemental materials.

#### An Unconditional Latent Curve/Multilevel Model

**Fig. 3:**
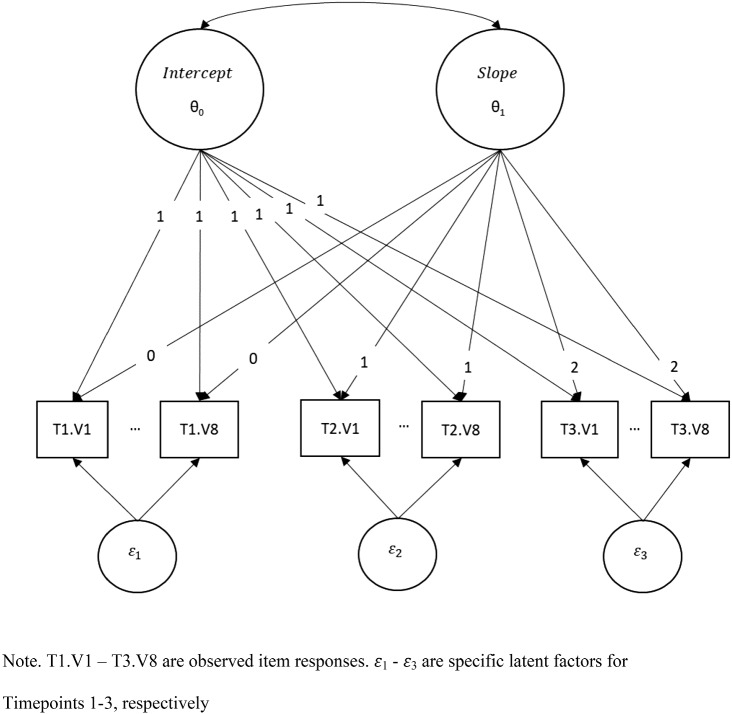
Structural model of a generic latent growth curve with three timepoints.

**Fig. 4:**
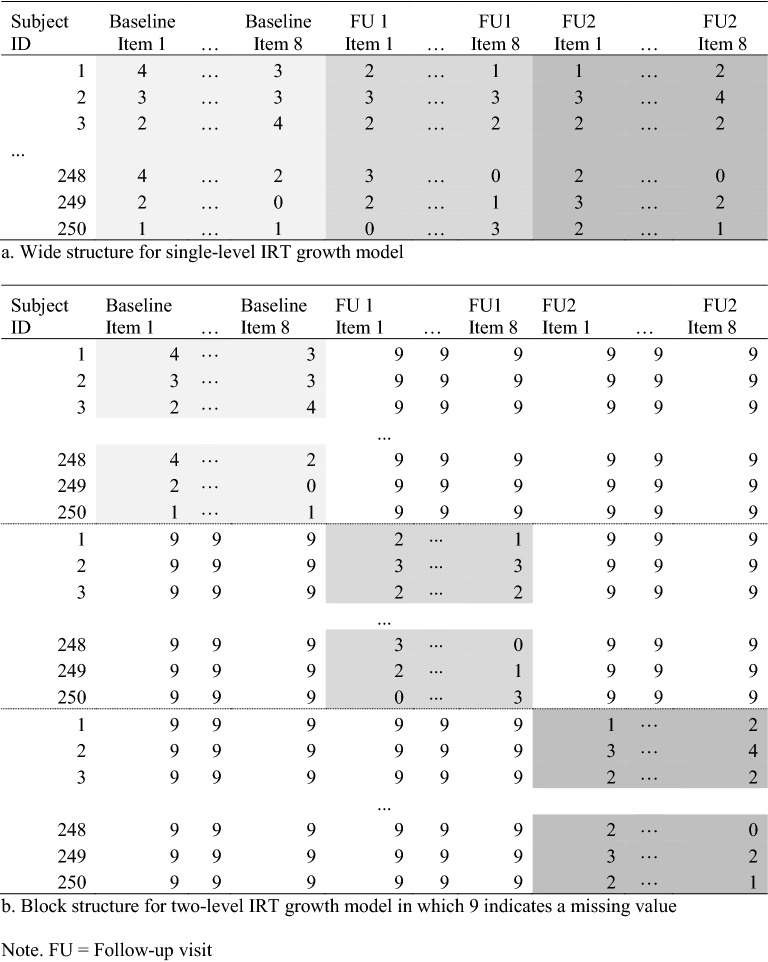
Data structures used in the latent variable model analyses.

The first and most obvious model that comes to mind when discussing change in the latent variable framework is the latent curve model (e.g., Meredith & Tisak, 1990) as specified in Eq. (4). Figure 3 provides a graphical representation of such a model. For this model, our data set is structured in the typical “wide” format, in which each observation is a unique individual and individual items at each time point are in columns (Fig. 4a). In the model, the latent growth model is specified as having five dimensions/factors. The first two will be used to define the Intercept ($\theta _{0})$ and Slope ($\theta _{1})$ latent variables (similar to Fig. 3). The remaining three ($\epsilon _{1},\epsilon _{2},\epsilon _{3})$ are timepoint-specific factors, used to address residual variability remaining between the PROMIS Short Form V.1.0-Sleep Disturbance 8aitems within each timepoint, with only items from a given timepoint loading on a specific factor. In this simple model, we omit the item-specific random effect $\xi$ for the ease of illustration. An important point to note is that the Intercept and Slope latent variables are in fact “general” dimensions in a two-tier model setup, with the residual terms ($\epsilon _{1},\epsilon _{2},\epsilon _{3})$ group/specific dimensions as in a bifactor (e.g., Gibbons & Hedeker, 1992) or, more generally, a two-tier model. With the item parameters set at the PROMIS banked values, we also freely estimate the means of Intercept and Slope factors, as well as their variances and covariance. These estimates (top half of Table 3) characterize the study sample relative to the PROMIS scale-setting population for the specific outcome of interest.

To illustrate that the equivalence between latent curve and multilevel growth models continues to hold even in a setting where the outcome variable is also latent, we now reparameterize the five-factor single-level IRT model into a three-factor multilevel IRT model, in which two of the factors are at Level-2 (“between”) and represent the Intercept and Slope random effects and the third factor is at Level-1. To fit this model, however, we need to restructure the data from “wide” format to what we refer to as a “block” format (Fig. 4b), in which each subject is represented in the dataset as many time as there are timepoints (in our case three), and while all observations have columns for all items by visit, observed responses for a given timepoint are only present on the rows associated with that timepoint. Restructuring the data in this way allows us to, in essence, collapse the three time-specific factors used in the single-level model so they are represented on a single factor.

**Table 3:** Single-level and multilevel latent variable growth model estimates.

Single-level growth model estimates					
Parameter	Intercept	Slope	Baseline residual	Follow-up 1 residual	Follow-up 2 residual
Mean (SE)	1.34 (0.07)	$-$ 0.55 (0.05)	0 (–)	0 (–)	0 (–)
Covariance matrix	0.15 (0.06)				
	0.07 (0.02)	0.05 (0.03)			
	0 (–)	0 (–)	1.00 (–)		
	0 (–)	0 (–)	0 (–)	1.00 (–)	
	0 (–)	0 (–)	0 (–)	0 (–)	1.00 (–)

Multilevel growth model estimates					
	Intercept	Slope	Residual		
Mean (SE)	1.34 (0.07)	$-$ 0.55 (0.05)	0 (–)		
Covariance matrix	0.15 (0.06)				
	0.07 (0.02)	0.05 (0.03)			
	0 (–)	0 (–)	1 (–)		

As with the first parameterization, the item parameters have been fixed to PROMIS item bank values and the only estimated parameters are the Level-2 latent variable means, variances, and covariance (bottom half of Table 3). The model is estimated with the BAEM algorithm. As can be seen from the observed estimated values, the single-level and multilevel model parameter point estimates are exactly the same as are the negative 2 log-likelihood, AIC and BIC (not shown; full results available in online supplemental materials), further demonstrating that these two are equivalent parameterizations.

This observation of equivalence is important, even though it is a side note to the main line of development in this paper, because it tells us that *bifactor/testlet/ two-tier type of (single-level) “hierarchical” item factor models have been multilevel IRT models all along*. The “hierarchical” factor pattern enables dimension reduction within a level. With the ability of modern IRT software to handle both multilevel data and these “hierarchical” models at the same time, one can already fit the multilayered models that Jeon et al. (2018) developed in a computationally efficient manner.

When parameterized as a single-level model, one benefit is that modern limited-information fit statistics (e.g., Maydeu-Olivares & Joe, 2005) are immediately available. The statistics suggest that the linear latent growth /multilevel model provided a very poor fit to the observed data ($M_{{2}}$-based $\hbox {RMSEA} = 0.09$). While some of the poor fit may be attributable to the reported models not including information about treatment assignment (which could be added), a larger issue with this growth model is the assumption of linearity over time. As seen in Fig. 1, the observed PROMIS Short Form V.1.0-Sleep Disturbance 8a*T*-scores are not decreasing at a constant rate over the course of the trial. Rather, there is a noticeable decline in scores from Baseline to Follow-up 1 and then a less steep decline from Follow-up 1 to Follow-up 2. It is not unreasonable to assume that the trends in the latent variables would follow a similar pattern, and this suggests that moving to a model that does not assume linear change over the course of the trial would be wise.

#### A Two-Tier Model

**Fig. 5:**
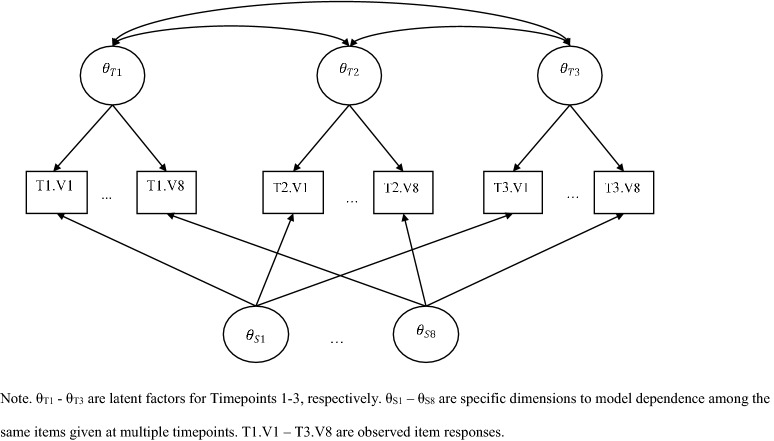
Structural model diagram for a two-tier, longitudinal model with 3 timepoints and 8 items per timepoint.

To address the concern regarding the assumption of linear change over time, we move to a two-tier model (Cai, 2010a), which models our three timepoints as individual factors and, per the suggestions of Paek et al. (2014), also includes specific factors for each of the eight PROMIS Short Form V.1.0-Sleep Disturbance 8a items to account for the residual dependence of responses to the same items over repeated visits. The basic form of this model is presented graphically in Fig. 5, and the factor pattern matrix for such a model is presented in Table 4. This model has a total of 11 (3 timepoint/general $+$ 8 item/specific) dimensions, which would typically be computationally impractical using the BAEM algorithm due to the so-called curse of dimensionality. However, because of the dimension reduction capabilities of the two-tier model (Cai, 2010a) when models conform to certain specifications (which this model does), the total dimension of integration required to estimate this model with BAEM is 4, rather than 11.

**Table 4:** Factor pattern matrix of the fitted two-tier latent variable model.

Item ID	Baseline	Follow-up 1	Follow-up 2	Item 1	Item 2	Item 3	Item 4	Item 5	Item 6	Item 7	Item 8
t1_item1	l1			l9							
t1_item2	l2				l10						
t1_item3	l3					l11					
t1_item4	l4						l12				
t1_item5	l5							l13			
t1_item6	l6								l14		
t1_item7	l7									l15	
t1_item8	l8										l16
t2_item1		l1		l9							
t2_item2		l2			l10						
t2_item3		l3				l11					
t2_item4		l4					l12				
t2_item5		l5						l13			
t2_item6		l6							l14		
t2_item7		l7								l15	
t2_item8		l8									l16
t3_item1			l1	l9							
t3_item2			l2		l10						
t3_item3			l3			l11					
t3_item4			l4				l12				
t3_item5			l5					l13			
t3_item6			l6						l14		
t3_item7			l7							l15	
t3_item8			l8								l16

l1–l8 fixed at PROMIS calibration values. l9–l16 freely estimated.

The slope values for all 11 latent variables (both fixed and estimated parameters) are presented in the top half of Table 5. The estimated latent variable means and covariance matrix values from this model are presented in the top half of Table 6. The estimated latent variable means have a ready interpretation (relative to a standard normal distribution [$\hbox {M} = 0$, $\hbox {SD} = 1$]), the latent SlpDist scores derived from the PROMIS items are rather high at Baseline (almost 1.4 SDs over the population mean), indicating significant sleep disturbance in the sample, and decrease over the course of the trial. Standardizing the covariance values reported in the lower section of Table 6, we find the correlation between the latent variable at Baseline and at Follow-up 1 is 0.39, between Baseline and Follow-up 2 is 0.31, and between Follow-up 1 and Follow-up 2 is 0.85; this supports the previously discussed issue with nonlinear change and highlights that the majority of the change/improvement that occurs over the course of the trial happens between Baseline and Follow-up 1 with patients’ SlpDist relatively more stable from Follow-up 1 to Follow-up 2.

**Table 5:** Slope parameter estimates and SEs for a two-tier latent variable model by estimation method.

Item ID	General Slope	Item 1 Slope	Item 2 Slope	Item 3 Slope	Item 4 Slope	Item 5 Slope	Item 6 Slope	Item 7 Slope	Item 8 Slope
*Results from BAEM estimation*									
Item 1	3.39	0.96 (0.15)							
Item 2	2.58		1.16 (0.13)						
Item 3	2.80			0.00 (0.38)					
Item 4	2.51				1.14 (0.13)				
Item 5	2.30					0.64 (0.15)			
Item 6	2.47						0.33 (0.26)		
Item 7	2.37							1.17 (0.13)	
Item 8	2.77								1.33 (0.14)
*Results from MH-RM estimation*									
Item 1	3.39	0.99 (0.12)							
Item 2	2.58		1.64 (0.11)						
Item 3	2.80			− 0.07 (0.45)					
Item 4	2.51				1.13 (0.12)				
Item 5	2.30					0.62 (0.20)			
Item 6	2.47						0.38 (0.47)		
Item 7	2.37							1.16 (0.11)	
Item 8	2.77								1.32 (0.12)

Estimates are constrained to equality across timepoints (Baseline, Follow-up 1, Follow-up 2)—see Table 4—so estimates are only reported once per item in the interest of space. Full results are available in the online supplemental materials.

**Table 6:** Group parameter estimates and SEs for a two-tier latent variable model by estimation method.

Parameter	Baseline	Follow-up 1	Follow-up 2	Item 1	Item 2	Item 3	Item 4	Item 5	Item 6	Item 7	Item 8
Results from BAEM estimation											
Mean (SE)	1.43 (0.06)	0.60 (0.07)	0.35 (0.07)	0 (–)	0 (–)	0 (–)	0 (–)	0 (–)	0 (–)	0 (–)	0 (–)
Covariance Matrix	0.73 (0.08)										
	0.33 (0.07)	0.96 (0.11)									
	0.28 (0.07)	0.89 (0.10)	1.13 (0.12)								
	0 (–)	0 (–)	0 (–)	1 (–)							
	0 (–)	0 (–)	0 (–)	0 (–)	1 (–)						
	0 (–)	0 (–)	0 (–)	0 (–)	0 (–)	1 (–)					
	0 (–)	0 (–)	0 (–)	0 (–)	0 (–)	0 (–)	1 (–)				
	0 (–)	0 (–)	0 (–)	0 (–)	0 (–)	0 (–)	0 (–)	1 (–)			
	0 (–)	0 (–)	0 (–)	0 (–)	0 (–)	0 (–)	0 (–)	0 (–)	1 (–)		
	0 (–)	0 (–)	0 (–)	0 (–)	0 (–)	0 (–)	0 (–)	0 (–)	0 (–)	1 (–)	
	0 (–)	0 (–)	0 (–)	0 (–)	0 (–)	0 (–)	0 (–)	0 (–)	0 (–)	0 (–)	1 (–)
Results from MH-RM estimation											
Mean (SE)	1.43 (0.06)	0.60 (0.07)	0.35 (0.07)	0 (–)	0 (–)	0 (–)	0 (–)	0 (–)	0 (–)	0 (–)	0 (–)
Covariance Matrix	0.75 (0.07)										
	0.34 (0.06)	0.99 (0.09)									
	0.29 (0.07)	0.90 (0.09)	1.16 (0.11)								
	0 (–)	0 (–)	0 (–)	1 (–)							
	0 (–)	0 (–)	0 (–)	0 (–)	1 (–)						
	0 (–)	0 (–)	0 (–)	0 (–)	0 (–)	1 (–)					
	0 (–)	0 (–)	0 (–)	0 (–)	0 (–)	0 (–)	1 (–)				
	0 (–)	0 (–)	0 (–)	0 (–)	0 (–)	0 (–)	0 (–)	1 (–)			
	0 (–)	0 (–)	0 (–)	0 (–)	0 (–)	0 (–)	0 (–)	0 (–)	1 (–)		
	0 (–)	0 (–)	0 (–)	0 (–)	0 (–)	0 (–)	0 (–)	0 (–)	0 (–)	1 (–)	
	0 (–)	0 (–)	0 (–)	0 (–)	0 (–)	0 (–)	0 (–)	0 (–)	0 (–)	0 (–)	1 (–)

While we were able to fit the two-tier model with BAEM under dimension reduction, the four-dimensional integration is near the practical limit of what can be efficiently estimated using this method. If additional timepoints were to be included in the model, BAEM would become computationally burdensome. To estimate truly high-dimensional MIRT models, it is necessary to switch to more recently developed estimation methods (e.g., Cai, 2010b, c; Edwards, 2010) that eschew multidimensional numerical integration. However, these new algorithms are less well-known and shrouded in more mystery. We hope to dispel some of the mystery here.

In the lower half of Tables 5 and 6, we have re-estimated the same two-tier model with the MH-RM algorithm. As one can see, both the point estimates and SEs of the estimated item and group parameters are extremely similar across the two estimation methods. We run this replication to provide further assurance that a stable solution is being obtained and that a more modern method such as MH-RM does provide optimization results on par with the venerable BAEM algorithm. Crucially, MH-RM takes about 10% of the time required for BAEM to reach convergence for this 11-dimensional model. The largest differences across the two estimation methods are for the estimated item-specific slope values for Item 3; in the BAEM results, this slope value appears to poorly estimated (as evidenced by the larger SE) in any case. While producing comparable maximum likelihood solutions, the move to newer estimation methods, as a key benefit in addition to speed, affords us additional flexibility in fitting high-dimensional models.

An interesting question to ask at this moment is whether the more complex model necessarily leads to improvements in model fit relative to the models in Sect. 4.2.1. With BAEM, log-likelihood-based fit indices are readily available and may be used to compare models. The unconditional growth models described in Sect. 4.2.1 have a $-\,2\times$ log-likelihood value of 13,810.51 ($\hbox {AIC} = 13{,}820.51$, $\hbox {BIC} = 13838.12$). The two-tier model described above has a $-\,2\times$ log-likelihood of 13334.19 ($\hbox {AIC} = 13{,}368.19$, $\hbox {BIC} = 13{,}428.06$). This represents a substantial improvement in model fit, while taking into account additional model complexity.

With the MH-RM algorithm, it is effortless to build on the previously fit two-tier model by incorporating treatment groups as coded design variables. The study contains placebo and active treatment. A reasonable approach would be to use a coded variable ($$x_{j})$$ to represent treatment group membership, with the omitted group (the placebo condition) serving as the reference cell. Using this formulation, we can obtain estimates of the degree to which treatment group influences each of the visit-specific latent variables in the form of regression coefficients. While full results (item and group parameters) are available in the online supplemental materials, Table 7 provides the estimated group parameters from this model. Because of the inclusion of treatment assignment indicator, the reported latent variable means have effectively become intercepts, representing the reference cell.

**Table 7:** Estimates and SEs for a two-tier latent variable model including treatment indicator from MH-RM estimation.

Parameter		Baseline	Follow-up 1	Follow-up 2	Item 1	Item 2	Item 3	Item 4	Item 5	Item 6	Item 7	Item 8
Latent mean (SE)		1.38 (0.06)	0.79 (0.06)	0.57 (0.07)	0 (–)	0 (–)	0 (–)	0 (–)	0 (–)	0 (–)	0 (–)	0 (–)
Regression coefficient (SE)	Tx	0.13 (0.08)	$-$ 0.43 (0.09)	$-$ 0.49 (0.10)								
Covariance matrix value (SE)		0.74 (0.07)										
		0.35 (0.06)	0.94 (0.09)									
		0.30 (0.07)	0.84 (0.09)	1.09 (0.10)								
		0 (–)	0 (–)	0 (–)	1 (–)							
		0 (–)	0 (–)	0 (–)	0 (–)	1 (–)						
		0 (–)	0 (–)	0 (–)	0 (–)	0 (–)	1 (–)					
		0 (–)	0 (–)	0 (–)	0 (–)	0 (–)	0 (–)	1 (–)				
		0 (–)	0 (–)	0 (–)	0 (–)	0 (–)	0 (–)	0 (–)	1 (–)			
		0 (–)	0 (–)	0 (–)	0 (–)	0 (–)	0 (–)	0 (–)	0 (–)	1 (–)		
		0 (–)	0 (–)	0 (–)	0 (–)	0 (–)	0 (–)	0 (–)	0 (–)	0 (–)	1 (–)	
		0 (–)	0 (–)	0 (–)	0 (–)	0 (–)	0 (–)	0 (–)	0 (–)	0 (–)	0 (–)	1 (–)

The newly added regression coefficients are the contrasts between the active treatment condition vs. the placebo. Based on the coefficients and the error covariance matrix, one can also conduct an omnibus test of the effect of treatment (using a Wald Chi-square statistic) or any other linear hypothesis test with specialized contrasts. This could be especially useful in trials with multiple treatment conditions.

#### An Alternative Model with Latent Differences

An alternative model to examine the change over time in the SlpDist of the clinical trial subjects can be formulated along the lines of Eq. (10). This is similar to the model set out in Cai et al. (2016; Sect. 4.2), which they note is motivated by longitudinal models previously described (e.g., Bock & Bargmann, 1966; Embretson, 1991; McArdle, 2009). While there are still three latent variables representing our timepoints the meaning of the latent variables is different from the previous two-tier model. The variable at the first timepoint continues to set a baseline, while the remaining two timepoints are interpretable as latent deviations or differences from the previous timepoint. The factor pattern that allows for the latent variables to be interpreted in this fashion is provided in Table 8. The primary results of interest from the latent difference model are the estimated group parameters, presented in Table 9. While the baseline latent variable mean estimate (1.42) is similar to previous estimates from other models, the Follow-up 1 and Follow-up 2 mean values are noticeably different due to the alternative interpretation. Rather than describing the “average” state at Follow-up 1, the Follow-up 1 latent variable mean in this model describes the average difference from Baseline to Follow-up 1. Similarly, the Follow-up 2 mean is interpreted as the difference from Follow-up 1 to Follow-up 2, rather than status of SlpDist at Follow-up 2. Parameterizing the change in this way allows us to conduct more detailed examinations into change over time and the prediction of that change.

**Table 8:** Factor pattern of a latent difference model.

Item ID	Baseline	Follow-up 1	Follow-up 2	Item 1	Item 2	Item 3	Item 4	Item 5	Item 6	Item 7	Item 8
t1_item1	l1			l9							
t1_item2	l2				l10						
t1_item3	l3					l11					
t1_item4	l4						l12				
t1_item5	l5							l13			
t1_item6	l6								l14		
t1_item7	l7									l15	
t1_item8	l8										l16
t2_item1	l1	l1		l9							
t2_item2	l2	l2			l10						
t2_item3	l3	l3				l11					
t2_item4	l4	l4					l12				
t2_item5	l5	l5						l13			
t2_item6	l6	l6							l14		
t2_item7	l7	l7								l15	
t2_item8	l8	l8									l16
t3_item1	l1	l1	l1	l9							
t3_item2	l2	l2	l2		l10						
t3_item3	l3	l3	l3			l11					
t3_item4	l4	l4	l4				l12				
t3_item5	l5	l5	l5					l13			
t3_item6	l6	l6	l6						l14		
t3_item7	l7	l7	l7							l15	
t3_item8	l8	l8	l8								l16

l1–l8 are all fixed at PROMIS-calibrated slope values. l9–l16 are freely estimated.

**Table 9:** Group parameters and SEs from a latent difference model.

Parameter	Baseline	Follow-up 1	Follow-up 2	Item 1	Item 2	Item 3	Item 4	Item 5	Item 6	Item 7	Item 8
Latent Mean (SE)	1.42 (0.05)	$-$ 0.81 (0.06)	$-$ 0.25 (0.05)	0 (–)	0 (–)	0 (–)	0 (–)	0 (–)	0 (–)	0 (–)	0 (–)
Covariance matrix	0.66 (0.07)										
	0 (–)	0.96 (0.09)									
	0 (–)	0 (–)	0.34 (0.03)								
	0 (–)	0 (–)	0 (–)	1 (–)							
	0 (–)	0 (–)	0 (–)	0 (–)	1 (–)						
	0 (–)	0 (–)	0 (–)	0 (–)	0 (–)	1 (–)					
	0 (–)	0 (–)	0 (–)	0 (–)	0 (–)	0 (–)	1 (–)				
	0 (–)	0 (–)	0 (–)	0 (–)	0 (–)	0 (–)	0 (–)	1 (–)			
	0 (–)	0 (–)	0 (–)	0 (–)	0 (–)	0 (–)	0 (–)	0 (–)	1 (–)		
	0 (–)	0 (–)	0 (–)	0 (–)	0 (–)	0 (–)	0 (–)	0 (–)	0 (–)	1 (–)	
	0 (–)	0 (–)	0 (–)	0 (–)	0 (–)	0 (–)	0 (–)	0 (–)	0 (–)	0 (–)	1 (–)

#### Latent Difference Model with Site Random Effect

We now fully build out the model in Sect. 4.2.2 with predictors and other important design features. In addition to including a treatment assignment variable as a predictor, we also include information regarding clinical site (as this was a multinational, multisite trial). The participants available at Baseline were collected from 67 different sites, with sample size per site ranging from 1 to 12. To incorporate the site information into the model, we now add a single level-2 (site-level) factor to account for the possible between-site variability. This is the full model as shown in Eq. (10). In this model, the Baseline, Follow-up 1, and Follow-up 2 latent difference variables are at level-1 (individual-level). The level-1 Baseline latent variable can be interpreted as further deviations from the site random intercept, which itself represents Baseline deviations among sites from the grand mean. These level-1 latent variables are regressed on the reference-cell-coded treatment assignment dummy variable, as before, to obtain estimates of treatment effects.

**Table 10:** Group parameters and SEs from a latent difference model including treatment indicator and a site intercept.

Parameter	Tx	Site	Baseline	Follow-up 1	Follow-up 2	Item 1	Item 2	Item 3	Item 4	Item 5	Item 6	Item 7	Item 8
Latent mean (SE)		0.89 (0.05)	0.43 (0.05)	$-$ 0.55 (0.06)	$-$ 0.22 (0.04)	0 (–)	0 (–)	0 (–)	0 (–)	0 (–)	0 (–)	0 (–)	0 (–)
Regression coefficient (SE)	Tx		0.12 (0.09)	-0.56 (0.10)	-0.06 (0.07)								
Covariance matrix value (SE)		0.17 (0.07)											
		0 (–)	0.47 (0.06)										
		0 (–)	0 (–)	0.86 (0.10)									
		0 (–)	0 (–)	0 (–)	0.32 (0.04)								
		0 (–)	0 (–)	0 (–)	0 (–)	1 (–)							
		0 (–)	0 (–)	0 (–)	0 (–)	0 (–)	1 (–)						
		0 (–)	0 (–)	0 (–)	0 (–)	0 (–)	0 (–)	1 (–)					
		0 (–)	0 (–)	0 (–)	0 (–)	0 (–)	0 (–)	0 (–)	1 (–)				
		0 (–)	0 (–)	0 (–)	0 (–)	0 (–)	0 (–)	0 (–)	0 (–)	1 (–)			
		0 (–)	0 (–)	0 (–)	0 (–)	0 (–)	0 (–)	0 (–)	0 (–)	0 (–)	1 (–)		
		0 (–)	0 (–)	0 (–)	0 (–)	0 (–)	0 (–)	0 (–)	0 (–)	0 (–)	0 (–)	1 (–)	
		0 (–)	0 (–)	0 (–)	0 (–)	0 (–)	0 (–)	0 (–)	0 (–)	0 (–)	0 (–)	0 (–)	1 (–)

The estimated group parameters from this model are presented in Table 10. From the reported values, we can make several interesting inferences. First, on average the placebo and active treatment groups do not differ significantly at Baseline ($\hbox {beta} = 0.12$, $\hbox {SE}= 0.09$). Second, there is statistically significant variability between the sites (variance of the level-2 site factor $= 0.17$, $\hbox {SE} = 0.07$) in the initial level of SlpDist. Together with the Baseline individual-level variance component of 0.47, this translates into an intra-class correlation of 0.27, which is not trivial. Note that additionally, on the efficacy of treatment with regard to changes in SlpDist, there is a statistically significant decrease in SlpDist (improvement in sleep) from Baseline to Follow-up 1 in general, even for the placebo group (Follow-up 1 latent difference $\hbox {M} = -\,0.55$, $\hbox {SE} = 0.06$). Furthermore, the change is significantly predicted by active treatment group membership (beta $= -\,0.56$, $\hbox {SE}= 0.10$), relative to the placebo group. Finally, while the placebo group continues to significantly decline in SlpDist from Follow-up 1 to Follow-up 2 (Follow-up 2 latent difference $\hbox {M} = -\,0.22$, $\hbox {SE} = 0.04$), there is no appreciable additional improvement due to active treatment from Follow-up 1 to Follow-up 2 (regression coefficient is not significantly different from 0). Due to the use of MH-RM algorithm, the final model’s marginal log-likelihood must be approximated by Monte Carlo integration. Chib and Jeliazkov’s (2001) method was used here over 250 additional samples, resulting in a $-\,2\times$ log-likelihood of 12,763.35 with two-side 95% CI of (12,736.54,12,790.15). The corresponding 95% CIs for AIC and BIC can be derived similarly, with $\hbox {AIC} = (12{,}774.54,12{,}828.15)$ and $\hbox {BIC} = (12{,}841.45,12{,}895.06)$. Again, these represent substantial improvements over the models that do not take site effect or the study design into account.

With the availability of the regression coefficients and their error covariance matrix, one could also conduct Wald tests for any linear hypothesis. For example, if our trial had multiple treatment groups (say, 5 in a dose-finding study), at Baseline, the overall difference among treatment groups could be formulated as a 4 degrees-of-freedom Wald test of the hypothesis that the coefficients for the regression of $\theta _{ij1}$ on the treatment assignment dummy variables are null (supporting successfully randomization). Analogously, the Chi-square for overall treatment group differences in the latent difference outcome $\theta _{ij2}$ at Follow-up 1 or $\theta _{ij3}$ at Follow-up 2 could also be constructed and formally tested*.*

## Discussion

PRO measures that were developed with and can be scored using IRT methods are seeing increasing adoption in the clinical trial space. This includes PROMIS measures, as shown, but is not exclusive to PROMIS. The models presented here will work with patients’ item responses on any high-quality measure that has been calibrated with regular IRT (e.g., Keller et al., 2014; Wirth et al., 2016), or more restricted IRT models in the Rasch family. Furthermore, they provide familiar inferential statistical methods that are analogous to linear models for repeated-measures analysis of variance or linear mixed-effects models. These models are well within practical reach with modern IRT software.

Our goal is not to propose any fundamentally new models previously unseen in the statistical literature. The hope is that by providing a convenient overview that connects various modeling frameworks together, both theoretically and empirically, we may start a productive conversation between psychometricians and other researchers in regulatory science. Building such a bridge could also evoke further research collaborations on innovative statistical methods that can enhance the usefulness of PROs in clinical trials.

## Footnotes

1 (Sleep Disturbance_Sleep Related Impairment V1.0 banks.xlsx) dated 3-17-2015.
